# Improving the Recognition of Bamboo Color and Spots Using a Novel YOLO Model

**DOI:** 10.3390/plants14152287

**Published:** 2025-07-24

**Authors:** Yunlong Zhang, Tangjie Nie, Qingping Zeng, Lijie Chen, Wei Liu, Wei Zhang, Long Tong

**Affiliations:** 1 College of Optical, Mechanical and Electrical Engineering, Zhejiang A&F University, Hangzhou 311300, China; 17367075305@163.com (Y.Z.);; 2 Chongqing Academy of Forestry, Chongqing 401147, China; 3 Research Institute of Subtropical Forestry, Chinese Academy of Forestry, Hangzhou 311400, China

**Keywords:** deep learning, bamboo shoots, classification, YOLOv8-BS model, phenotype

## Abstract

The sheaths of bamboo shoots, characterized by distinct colors and spotting patterns, are key phenotypic markers influencing species classification, market value, and genetic studies. This study introduces YOLOv8-BS, a deep learning model optimized for detecting these traits in *Chimonobambusa utilis* using a dataset from Jinfo Mountain, China. Enhanced by data augmentation techniques, including translation, flipping, and contrast adjustment, YOLOv8-BS outperformed benchmark models (YOLOv7, YOLOv5, YOLOX, and Faster R-CNN) in color and spot detection. For color detection, it achieved a precision of 85.9%, a recall of 83.4%, an F1-score of 84.6%, and an average precision (AP) of 86.8%. For spot detection, it recorded a precision of 90.1%, a recall of 92.5%, an F1-score of 91.1%, and an AP of 96.1%. These results demonstrate superior accuracy and robustness, enabling precise phenotypic analysis for bamboo germplasm evaluation and genetic diversity studies. YOLOv8-BS supports precision agriculture by providing a scalable tool for sustainable bamboo-based industries. Future improvements could enhance model adaptability for fine-grained varietal differences and real-time applications.

## 1. Introduction

A cornerstone of sustainable agriculture, bamboo is a versatile resource widely distributed across China. It encompasses 47 genera, 770 species, 55 varieties, and 251 cultivated forms [1,2,3,4]. Bamboo is renowned for its rapid growth and high yields. It provides timber and edible shoots and contributes significantly to ecological stability and economic development [5,6]. Valued for their rich dietary fiber, protein, amino acids, polysaccharides, and minerals, bamboo shoots are a high-value commodity. The global market is projected to reach USD 702.97 million by 2032, growing at a compound annual rate of 6.5% [7,8,9,10,11,12]. This study focuses on *Chimonobambusa utilis*, a rare species native to the Dalou Mountain Range spanning Tongzi County in Guizhou and Jinfeng Mountain in Chongqing. These shoots grow at altitudes of 1400–2000 m and are harvested from August to October. They are prized for their exceptional freshness, aroma, tenderness, and crispness, and command premium prices in domestic and international markets [13,14,15]. Improvements in transportation infrastructure have increased their market reach and consumer demand, highlighting their potential for genetic and commercial development [16,17,18].

The color and spotting patterns of bamboo shoot sheaths are critical phenotypic traits, serving as indicators of environmental conditions, health status, harvest timing, and disease susceptibility [19,20]. For instance, irregular black, white, or brown spots may result from physical stress, microbial infections, or enzymatic changes during aging, which affect shoot quality [21]. Sheath pigmentation also influences edibility and is potentially regulated by light signaling pathways [22]. In *C. utilis*, these traits are particularly significant due to the species’ limited varietal diversity and untapped germplasm potential [23]. Accurate identification of sheath colors and spots is essential for genetic breeding, quality assessment, and ecological adaptability studies, which support the development of high-quality varieties [24]. However, only a limited number of new *C. utilis* varieties from Jinfo Mountain have been documented, highlighting the necessity of advanced phenotyping methods to explore and utilize its genetic resources [23].

Traditional methods of quantifying bamboo shoot phenotypic traits rely heavily on manual identification, presenting significant challenges. Prolonged repetitive work leads to worker fatigue and reduced accuracy. Overlapping sheaths in clustered bamboo shoots also complicate the differentiation of color and spot characteristics. These factors result in error rates of 15–25% [10]. Furthermore, manual identification is inefficient, often requiring several hours to assess a single batch of shoots, rendering it impractical for large-scale plantations or research projects [25]. Physical handling during manual inspection can damage shoots, compromising their quality and growth potential [25]. These limitations underscore the urgent need for automated, precise, and non-destructive methods to meet the growing demand for accuracy in bamboo shoot phenotyping.

Deep learning, a transformative branch of machine learning, has revolutionized image processing tasks by enabling the automated extraction of features from large-scale datasets [26,27,28]. Convolutional neural networks (CNNs) are widely used because they can extract complex features and perform accurate classification without manual feature engineering [29,30,31,32]. In agriculture, deep learning has been applied to tasks such as classifying crop varieties, detecting pests and diseases, assessing fruit maturity, and predicting yields [33,34,35,36]. The development of object detection algorithms has further deepened their application. Among the mainstream object detection algorithms in deep learning, Faster R-CNN (Region-based Convolutional Neural Network) serves as a significant milestone [37]. By introducing the Region Proposal Network (RPN), which shares convolutional features with the detection network, this algorithm significantly enhances the efficiency of object proposal generation and detection speed [38]. Its successful application in agricultural object detection is demonstrated by the effective identification of crops, pests, and agricultural facilities through precise boundary delineation [39]. Despite its high accuracy, however, its relatively high computational requirements limit its applicability in real-time scenarios.

The YOLO (You Only Look Once) series is known for its real-time object detection capabilities. It has been particularly impactful in identifying agricultural targets with high accuracy while reducing labor and time costs [40,41]. YOLOX [42] adopts an Anchor-free design and integrates the SimOTA dynamic label assignment strategy, simplifying the detection process while improving localization accuracy. It shows advantages in detecting irregular targets such as spots on bamboo shoot sheaths, though its inference speed for high-resolution images is slightly lower, which may hinder real-time performance in large-scale phenotyping analysis. YOLOv5 [43] uses CSPDarknet53 as its backbone and fuses multi-scale features via PANet, achieving an accuracy of up to 91.5% in agricultural pest and disease detection [44,45,46], yet with limited capability in handling scenarios where the target and background have similar colors—a common situation in bamboo shoot sheath detection. YOLOv7 [47] incorporates the ELAN structure and a compound scaling strategy to enhance feature learning and task adaptability, performing better than previous versions in detecting small agricultural pests but being less effective in identifying subtle color differences of bamboo shoot sheaths due to the lack of optimization for color feature extraction. The latest iteration, YOLOv8, has shown exceptional performance in crop yield prediction, achieving error rates below 5% for rice panicles and apple fruits. It is 40% more efficient and 15% more accurate than manual methods [48,49,50]. These advancements highlight the potential of deep learning to overcome complex phenotyping challenges in agriculture [51].

Despite technological advances, accurately quantifying bamboo shoot phenotypic traits, especially sheath colors and spots, remains challenging. Current methods, including traditional image analysis and early deep learning approaches, have difficulty with the complexity of natural environments and the subtle morphological differences among bamboo shoot varieties [52]. To address this issue, we developed YOLOv8-BS, a specialized model based on the YOLOv8 framework that is tailored to detect and classify the color and spotting patterns of *C. utilis* sheaths. This study aims to bridge the gap between technological innovation and practical applications in bamboo shoot cultivation by leveraging advanced image processing and deep learning techniques. The aims of this study are (1) to develop a high-precision color recognition model for individual bamboo shoots to provide reliable data for phenotypic and genetic studies, and (2) to create an efficient method for detecting sheath spots to enhance precision agriculture and support the sustainable development of bamboo-based industries.

## 2. Materials and Methods

The YOLO series has evolved through multiple iterations, with YOLOv8 emerging as the most advanced version to date, offering optimized detection performance across various tasks. This version includes several variants—YOLOv8n, YOLOv8s, YOLOv8m, YOLOv8l, and YOLOv8x—differentiated by network depth and width. Among these, YOLOv8s stands out for its deep architecture, delivering high-precision detection while maintaining low storage requirements and rapid inference speeds, making it ideal for resource-constrained environments such as remote sensing applications in agriculture.

**Figure 1:**
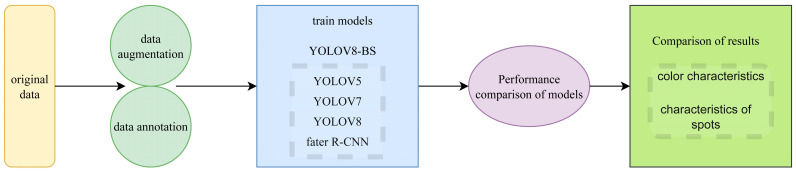
Technical route.

Given these attributes, YOLOv8-BS was developed based on the YOLOv8s architecture, enhancing its capability to process high-resolution imagery for bamboo shoot phenotyping. This adaptation is particularly suited for integrating with remote sensing technologies, where efficient analysis of field-collected data is critical for precision agriculture. The technical route, illustrated in Figure 1, outlines the workflow from data preparation to model training and performance evaluation, providing a structured approach to achieving accurate and rapid phenotyping. This includes data augmentation and annotation to enhance model robustness, followed by training against benchmark models (YOLOv7, YOLOv5, YOLOX, and Faster R-CNN), and a comparative analysis of results focusing on color and spot characteristics.

### 2.1. Experiment Location

**Figure 2:**
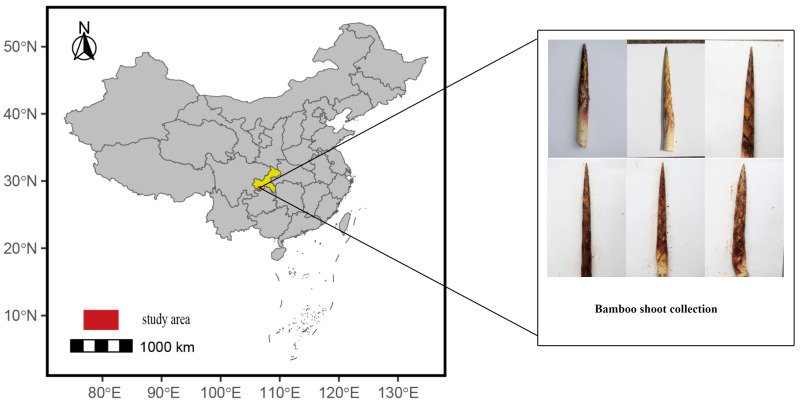
Image collection area.

This study was conducted at Jinfo Mountain in the Nanchuan District of Chongqing, China (29.023458° N, 107.193645° E), as shown in Figure 2. The region is characterized by a temperate monsoon climate with rugged terrain, elevations ranging from 1500 to 2000 m, annual rainfall from 1200 to 2000 mm, a frost-free period of about 120 days, and average temperatures between 12 and 15 °C. These conditions create a unique microhabitat for *C. utilis*, influencing the phenotypic diversity of bamboo shoot sheaths, particularly their color and spotting patterns. The study area was selected to capture a representative range of environmental and phenotypic variations, ensuring a diverse dataset for model training.

### 2.2. Selection of Bamboo Shoot Samples and Sampling Point Setup

The image sampling of *C. utilis* shoot sheaths was conducted at Jinfo Mountain from August to October 2024, which coincided with the species’ peak shooting period. During this time, new shoots continuously germinate, and their sheaths remain morphologically stable—neither lignified nor shed—thus fully preserving key phenotypic features such as texture, color variations, and spotting patterns. In contrast, during other seasons (e.g., winter), bamboo shoots enter a dormant state, and their sheaths are prone to aging and degradation, which does not meet the analytical requirements. To ensure consistency and reliability, only young shoots (2–4 weeks post-germination) were selected for sampling, as their sheaths, having unfolded and retained a tender texture, exhibit clear and stable color and spotting characteristics. Older shoots (over 5 weeks post-germination) were excluded, as their sheaths tend to brown and crack, introducing potential interference. To ensure the comprehensiveness of the samples, 120 representative sampling points were selected through field surveys, covering various spatial distributions, color variations, and spotting phenotypes. For each sampling point, geographic coordinates, surrounding vegetation, and environmental characteristics were recorded.

### 2.3. Collection and Preprocessing of the Dataset

The image acquisition was carried out using a Canon EOS 70D camera (24 megapixels, with a resolution of 6000 × 4000 pixels; Canon, Tokyo, Japan), which enabled the precise capture of phenotypic details such as texture and subtle spotting patterns. To ensure the consistency of visual data, strict control measures were implemented: image acquisition was limited to morning and evening under diffuse light conditions (avoiding strong direct sunlight) to minimize the impact of lighting fluctuations; the auto white balance (AWB) mode was uniformly adopted to adapt to subtle changes in illumination (e.g., variations in cloud cover) and stabilize the color features of the sheaths; the shooting distance was standardized to 30–50 cm, ensuring that the sheath occupied no less than 60% of the frame (facilitating subsequent cropping) while avoiding edge blurring caused by proximity to the camera’s minimum focusing distance (approximately 25 cm). Operators maintained distance consistency through a standardized visual alignment process via the viewfinder. Camera parameters were also dynamically optimized based on environmental conditions (aperture f/5.6–8.0, focal length 50–100 mm, ISO 100–400) to enhance the clarity of details. A total of over 2000 images were collected, covering all major phenotypic categories. To support model training, these images underwent preprocessing, including cropping to isolate sheath regions, lighting normalization through histogram equalization, and resizing to 640 × 640 pixels to meet the input requirements of YOLOv8-BS. Data augmentation techniques such as translation, horizontal flipping, random cropping, and contrast adjustment were applied to improve the robustness of the dataset and mitigate overfitting. The final dataset was split into a training set (1400 images) and a test set (600 images) at a ratio of 7:3, ensuring a balanced distribution of color and spotting categories in both sets. The image sampling area is shown in Figure 2.

### 2.4. Dataset Annotation

Accurate dataset annotation was essential for training and evaluating YOLOv8-BS. The annotation process involved classifying sheath colors into five categories (purple, brown, black, yellow, and green) and categorizing patterns as either spotted or unspotted. Over 2000 images were annotated using LabelMe (version 5.3.1) [53], generating more than 4000 bounding boxes to delineate target regions for model training. Challenges in the annotation process included occlusion by vegetation, inter-rater variability, and subtle phenotypic similarities among sheaths. To address these challenges, we have established a rigorous standardized process. In the preparatory stage, we first formulated classification criteria for colors and spots. For each color category, we defined the RGB color gamut range. For example, the purple category is defined as R (120–255), G (0–150), B (120–255), and physical photos with different brightness levels are provided as references. Regarding spots, it is specified that if there are three or more heterochromatic patches with a diameter of ≥1 mm and a total area ratio of ≥5% on the surface of a single sheath, it is classified as “spotted”; otherwise, it is classified as “spotless”. In the annotation implementation phase, operations adhered rigorously to predefined standards. Annotators were required to perform self-reviews against standardized atlases after completing every 200 images. Simultaneously, a dual-layer quality control mechanism was instituted: following the initial annotation, a second independent annotator conducted cross-validation to identify discrepancies, errors, or omissions. This iterative peer-review process enabled systematic error detection and correction, thereby enhancing the dataset’s overall veracity. While no annotation strategy can guarantee perfect accuracy, this framework significantly mitigated inter-rater variability and reduced annotation error rates, establishing a robust foundation for downstream analytical reliability.

### 2.5. YOLOv8-BS Model Architecture

**Figure 3:**
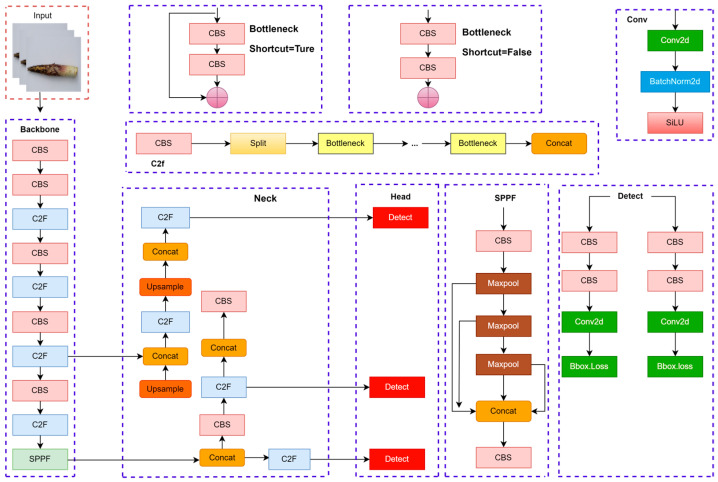
Schematic diagram of the network structure of YOLOv8.

The YOLOv8-BS model, derived from the YOLOv8s framework [54], is a fully convolutional neural network designed for precise phenotyping of bamboo shoot sheaths using high-resolution imagery. Optimized for detecting sheath colors and spotting patterns, the model comprises three core components: a backbone for feature extraction, a neck for feature aggregation, and a detection head for object prediction. Figure 3 illustrates the architecture, depicting the interconnections among these components to process bamboo shoot images effectively. The backbone, built on the YOLOv8s architecture, integrates the C2f (Cross-Stage Partial Fusion) module, an advancement of the CSPNet framework [55]. The C2f module enhances feature extraction depth and expressiveness while reducing computational complexity, making it suitable for high-resolution inputs. Additionally, the backbone employs the RepConv (Re-parameterizable Convolution) module, which uses a multi-branch structure during training to improve nonlinear modeling [56]. At inference, RepConv consolidates into a single branch, balancing efficiency and performance for real-time applications, such as field-based phenotyping. The neck module aggregates multi-scale features from the backbone to enhance detection accuracy across diverse bamboo shoot phenotypes. It incorporates three key components: (1) the Spatial Pyramid Pooling Fast (SPPF) module, which applies multi-scale pooling (e.g., 5 × 5, 9 × 9, 13 × 13 kernels) to capture features from varying sheath sizes, improving robustness in complex field conditions [57]; (2) the Probabilistic Anchor Assignment (PAA) module, which refines sample selection to optimize anchor box assignments, enhancing training efficiency [58]; and (3) the Path Aggregation Network (PAN), which fuses low-level spatial details with high-level semantic information via bidirectional pathways, strengthening feature representation [59]. These components ensure accurate detection of subtle phenotypic variations, such as small spots or faint color gradients. The detection head employs an anchor-free design, directly predicting target center coordinates, bounding box dimensions, and class probabilities for sheath colors and spotting patterns. This approach eliminates the need for predefined anchor boxes, improving training stability and generalization across diverse bamboo shoot morphologies. The model uses the Complete IoU (CIoU) loss function, which accounts for bounding box overlap, aspect ratio, and center distance, to refine localization accuracy [60]. The CIoU loss was selected for its ability to handle the irregular shapes and sizes of bamboo shoot sheaths, ensuring precise phenotyping in remote sensing contexts.

### 2.6. Programming Environment

The development and training of the YOLOv8-BS model for bamboo shoot sheath phenotyping required a robust programming environment to handle high-resolution imagery and complex deep learning tasks. This study utilized Python 3.8.17 as the primary programming language due to its extensive libraries and compatibility with deep learning frameworks. PyCharm (version 2023.1.4) served as the integrated development environment (IDE), offering advanced debugging and code management tools to streamline model implementation. The Anaconda3 platform (version 2023.03) facilitated dependency management by creating a consistent environment with pre-installed packages, including NumPy and OpenCV, essential for image preprocessing and data handling.

The algorithms were implemented using PyTorch 1.7.1, a flexible deep learning framework optimized for dynamic computational graphs and GPU acceleration. PyTorch was paired with CUDA 11.0 to leverage GPU parallelization, significantly reducing training time for the YOLOv8-BS model. Key PyTorch libraries, such as torchvision 0.8.2, supported image transformations and model evaluation. All computations were performed on a high-performance system running Windows 10 Pro, configured to support PyTorch and CUDA compatibility. The system was equipped with a 13th-generation Intel Core i9-13900KF CPU, 128 GB of DDR5 RAM, and an NVIDIA GeForce RTX 4090 GPU with 24 GB of GDDR6X memory.

### 2.7. Model Training

The YOLOv8-BS model is designed to detect and classify the colors and spotting patterns of the sheaths of *C. utilis* bamboo shoots. The model was trained using a dataset consisting of 6186 images of *C. utilis* sheaths, as described in Section 2.3. It should be clarified that this dataset of 6186 images includes both original images and augmented data. Specifically, as mentioned in Section 2.3, 2062 original images were collected, preprocessed, and annotated. To expand the dataset size and enhance the robustness of the model, data augmentation techniques were applied to these original images, resulting in an augmented dataset with a total of 6186 images (including 2062 original images and 4124 augmented images). For ensuring the reliability of model development and evaluation, this augmented dataset was split into a training set (70%, 4330 images), a validation set (10%, 619 images), and a testing set (20%, 1237 images). The training process was conducted on the high-performance system detailed in Section 2.6. Training spanned 500 epochs with a batch size of 16 and used the Adam optimizer with an initial learning rate of 0.01 to facilitate convergence. A cosine annealing scheduler gradually reduced the learning rate to 0.0001, enhancing model stability. To mitigate overfitting, a weight decay of 0.0005 was applied, and data augmentation techniques (e.g., random flipping, scaling, and color jittering) were employed during training, as detailed in Section 2.3. The complete intersection over union (CIoU) loss function optimized bounding box regression. It was selected for its ability to handle the irregular shapes and varying sizes of bamboo shoot sheaths. This ensured precise localization of phenotypic features.

For benchmarking purposes, YOLOv8-BS was compared to YOLOv7, YOLOv5, YOLOX, and Faster R-CNN using the same dataset, hardware, and evaluation metrics. The models were trained under identical conditions (500 epochs and a resolution of 640 × 640) to ensure a fair comparison. Validation performance was monitored to implement early stopping if no improvement occurred after 50 epochs to preserve model generalization.

### 2.8. Model Evaluation Metrics

The performance of the YOLOv8-BS model in extracting bamboo shoot sheath colors and spotting patterns was assessed using multiple metrics: precision (P), recall (R), F1-score, average precision (AP), mean average precision (mAP), and Intersection over union (IoU). Precision measures the proportion of correctly predicted positive samples among all predicted positives, defined as:

(1) $$P=\frac{\mathrm{TP}}{\mathrm{TP}+\mathrm{FP}}$$

Recall quantifies the proportion of actual positive samples correctly identified:

(2) $$R=\frac{\mathrm{TP}}{\mathrm{TP}+\mathrm{FN}}$$

The F1-score, a harmonic mean of precision and recall, balances these metrics, particularly for imbalanced datasets:

(3) $$F1-\mathrm{Score}=2\times \frac{P\times R}{P+R}$$

AP evaluates the model’s precision across all recall levels by integrating the precision–recall curve:

(4) $$\mathrm{AP}=\int_{0}^{1}P\left(R\right)\mathrm{dR}$$

The mAP averages AP across all categories (five colors and two spotting patterns in this study):

(5) $$\mathrm{mAP}=\frac{\sum_{i=1}^{N}{\mathrm{AP}}_{i}}{C}$$

where $\;{\mathrm{AP}}_{i}\;$ is the AP for the *i*th category, and N is the total number of categories. Intersection over union (IoU) is a crucial metric in the field of object detection for measuring the degree of overlap between the model’s predicted bounding box and the ground truth bounding box. Here, A and B represent the areas covered by the model’s predicted bounding box and the ground truth bounding box, respectively. The numerator A∩B denotes the area of the overlapping region between the two boxes, while the denominator A∪B refers to the total area covered by the combined region of the two boxes. By calculating the ratio of these two values, the localization accuracy of the detection box can be quantitatively evaluated:

(6) $$\mathrm{IoU}=\frac{\left|A\cap B\right|}{\left|A\cup B\right|}$$

An IoU threshold of 0.5 was adopted to classify predictions as true positives, aligning with standard object detection practices [61]. These metrics collectively provide a comprehensive evaluation of the model’s classification and localization performance, ensuring robust assessment of its capability in bamboo shoot phenotyping tasks.

## 3. Results

### 3.1. Model Performance and Threshold Sensitivity

This study evaluated the performance of YOLOv8-BS against four benchmark object detection models (YOLOv7, YOLOv5, YOLOX, and Faster R-CNN) for identifying colors and patterns on *C. utilis* bamboo shoot sheaths. All models were trained under identical conditions and evaluated using P, R, the F1-score, and average precision (AP) at an IoU threshold of 0.5. The evaluation focused on two tasks: color detection (five categories: purple, brown, black, yellow, and green) and spot detection (two categories: spotted and unspotted). Performance metrics were analyzed at confidence thresholds of 0.5 and 0.75 to assess robustness.

YOLOv8-BS outperformed all benchmarks for color detection, achieving an AP of 86.8% at threshold = 0.5 with a precision, recall, and F1-score of 85.9%, 83.4%, and 84.6%, respectively (Table 1). YOLOv7 and YOLOv5 followed with APof 75.4% and 71.2%, respectively. YOLOX and Faster-RCNN lagged behind with AP of 58.2% and 48.3%, respectively. At threshold = 0.75, YOLOv8-BS maintained robust performance (AP = 73.2%, F1 = 73.4%), whereas Faster R-CNN’s AP dropped significantly to 31.9%. These results highlight YOLOv8-BS’s superior accuracy in capturing subtle color gradients under varying confidence levels.

In spot detection, YOLOv8-BS further excelled, achieving an AP of 96.1% at a threshold of 0.5 and recording precision, recall, and an F1-score of 90.1%, 92.5%, and 91.1%, respectively (Table 2). YOLOv7 and YOLOv5 achieved AP of 78.6% and 74.6%, respectively, while YOLOX and Faster R-CNN trailed with APof 53.4% and 49.2%, respectively. At threshold = 0.75, YOLOv8-BS’s AP remained high at 86.7%, demonstrating stability. Notably, all models performed better in spot detection than in color detection, likely because distinct spot patterns are more distinguishable than continuous color variations in the dataset. For example, YOLOv7′s AP increased from 75.4% for color to 78.6% for spots at threshold = 0.5. Threshold sensitivity analysis revealed varying model robustness. YOLOv8-BS exhibited the smallest performance decline as the threshold increased.

### 3.2. Color and Spot Detection Performance

**Figure 4:**
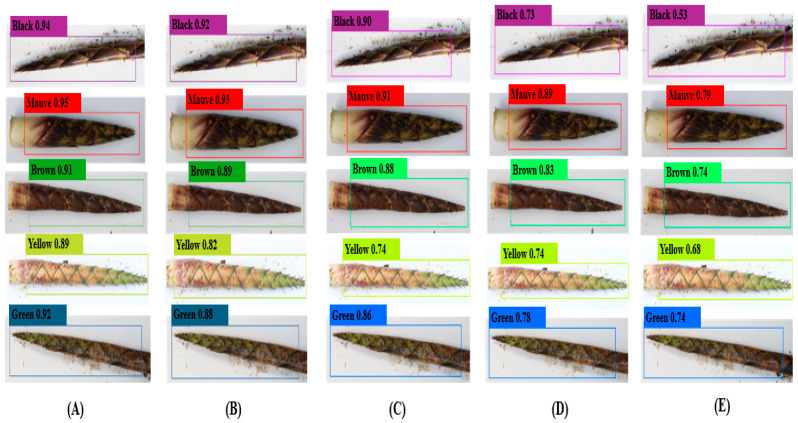
Detection confidence scores for bamboo shoot colors. Note: Panels (**A**) YOLOv8-BS, (**B**) YOLOv7, (**C**) YOLOv5, (**D**) YOLOX, and (**E**) Faster R-CNN, showing confidence scores at IoU threshold of 0.5.

**Figure 5:**
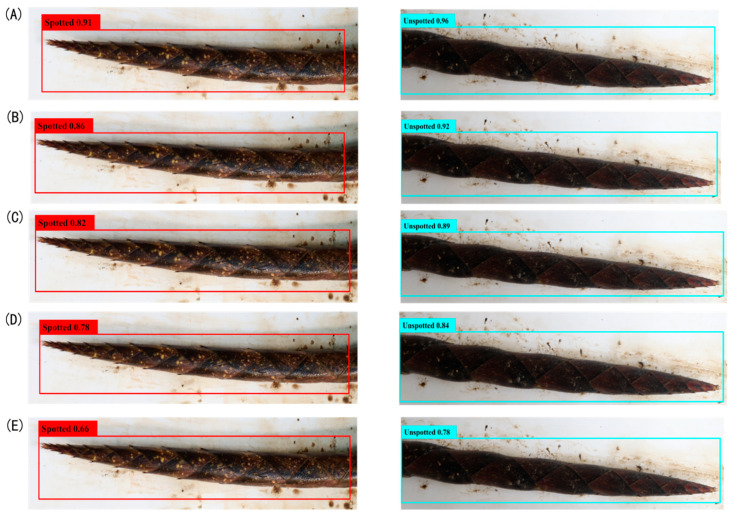
Detection confidence scores for bamboo shoot spots. Note: Panels (**A**) YOLOv8-BS, (**B**) YOLOv7, (**C**) YOLOv5, (**D**) YOLOX, and (**E**) Faster R-CNN, showing confidence scores at IoU threshold of 0.5.

The detection performance of the YOLOv8-BS model for identifying the colors and spots of *C. utilis* bamboo shoot sheaths was evaluated against the performance of YOLOv7, YOLOv5, YOLOX, and Faster R-CNN. Figure 4 and Figure 5 show the detection confidence scores for color and spot classification, respectively, at an IoU threshold of 0.5.

For color detection (Figure 4), YOLOv8-BS (A) exhibited the highest levels of confidence across categories, peaking at 0.94 for black bamboo shoots and achieving consistent scores above 0.85 for mauve, brown, yellow, and green (e.g., 0.89 for yellow). This reflects its superior target recognition, likely due to the optimized C2f and SPPF modules (Section 2.4). YOLOv7 (B) and YOLOv5 (C) showed moderate confidence levels (e.g., 0.90 for black in YOLOv7), whereas YOLOX (D) and Faster R-CNN (E) performed less well, with scores below 0.73 indicating weaker feature extraction in variable field conditions. The stability of YOLOv8-BS under high-confidence demands highlights its architectural advantage over the YOLO series and the traditional Faster R-CNN.

For spot detection (Figure 5), YOLOv8-BS (A) achieved confidence scores of 0.91 for spotted bamboo shoots and 0.96 for unspotted ones, demonstrating the precise differentiation enabled by its anchor-free design (Section 2.5). YOLOv7 (B) recorded 0.86 and 0.92 for spotted and unspotted, respectively, while YOLOv5 (C) and YOLOX (D) declined to 0.78 and 0.66. Faster R-CNN (E) showed the lowest scores (0.66 for spotted and 0.78 for unspotted), likely due to its limited adaptability to complex spot patterns. These results highlight the robustness of YOLOv8-BS, supporting its application in precision bamboo phenotyping.

### 3.3. Confusion Matrix Analysis

**Figure 6:**
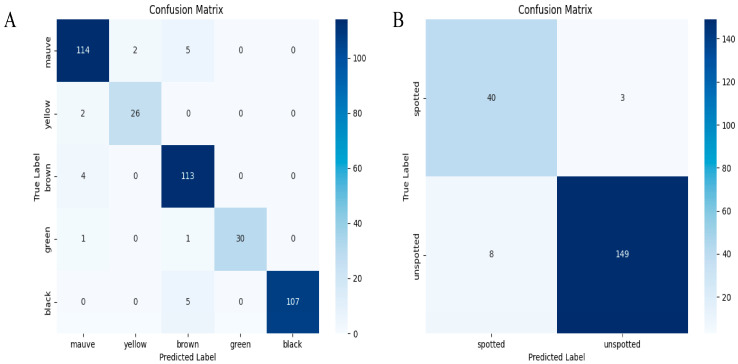
Confusion matrices for YOLOv8-BS. Note: (**A**) matrix for color detection (mauve, yellow, brown, green, black); (**B**) matrix for spot detection (spotted, unspotted), showing true vs. predicted labels with counts.

The classification accuracy of the YOLOv8-BS model was assessed using confusion matrices for color and spot detection, as shown in Figure 6. This provides a quantification of TP, FP, and FN across five color categories (mauve, yellow, brown, green, and black) and two spot categories (spotted and unspotted).

For color detection (left matrix), the diagonal elements indicate strong agreement between the predicted and true labels: 149 unspotted instances and 113 mauve instances were correctly classified. However, there were 40 misclassifications of spotted instances as unspotted and 8 misclassifications of unspotted instances as spotted, suggesting difficulty in distinguishing ambiguous phenotypes. Overall accuracy was 87.6%, calculated as the ratio of correct predictions to total instances, with a misclassification rate of 12.4%. For spot detection (right matrix), 313 brown instances and 107 black instances were accurately predicted, with only two and five misclassifications, respectively. This yielded an accuracy of 92.3% and a misclassification rate of 7.7%. This improvement is consistent with the distinct boundaries of spots versus gradual color transitions (Section 3.1).

## 4. Discussion

The YOLOv8-BS model, developed in this study, represents a specialized extension of the YOLOv8 framework, tailored for the detection and classification of bamboo shoot sheath colors and spots. As a derivative of the YOLO series, one of the leading object detection algorithms known for its balance of accuracy and speed through regression-based prediction, this model inherits the efficiency and adaptability of its predecessor while addressing the unique demands of botanical phenotyping. Bamboo shoots, particularly those of *C. utilis*, are a cornerstone of sustainable agriculture due to their ecological, nutritional, and economic value. The colors and spotting patterns of bamboo shoot sheaths are critical phenotypic traits that influence variety classification, health assessment, and market quality grading. The results demonstrate that the YOLOv8-BS model excels in detecting these traits, achieving AP values of 86.8% for color detection and 96.1% for spot detection. This surpasses the performance of benchmark models such as YOLOv7, YOLOv5, YOLOX, and Faster R-CNN. These results highlight the potential of the model to advance automated phenotyping in precision agriculture.

### 4.1. Model Performance Analysis

As detailed in the results section, the YOLOv8-BS model demonstrated superior performance in detecting and classifying the colors and spots of *C. utilis* bamboo shoot sheaths. For color detection, YOLOv8-BS achieved an average precision (AP) of 86.8% at an intersection over union (IoU) threshold of 0.5, with a precision, recall, and F1-score all exceeding 83.0% (Table 1). For spot detection, the model performed even better, achieving an AP of 96.1%, with respective precision, recall, and F1-scores of 90.1%, 92.5% and 91.1% (Table 2). These metrics significantly outperformed those of the benchmark models, including YOLOv7 (color AP: 75.4%; spot AP: 78.6%), YOLOv5 (color AP: 71.2%; spot AP: 74.6%), YOLOX (color AP: 58.2%; spot AP: 53.4%), and Faster R-CNN (color AP: 48.3%; spot AP: 49.2%). Figure 4 and Figure 5 further validated the model’s high confidence scores, e.g., 0.94 for black bamboo shoots and 0.96 for unspotted sheaths, compared to lower scores (e.g., 0.73 for Faster R-CNN in color detection).

These results highlight the capability of YOLOv8-BS to accurately and reliably identify key phenotypic traits, which are essential for variety classification, health monitoring, and quality assessment in precision agriculture [62]. The model’s high precision and recall minimize misclassifications, ensuring robust data for downstream analyses like genetic breeding. Compared to traditional two-stage detectors like Faster R-CNN, which rely on region proposal networks and exhibit lower efficiency in complex field conditions [63], YOLOv8-BS’s single-stage, anchor-free design significantly enhances detection speed and accuracy.

A notable observation is the model’s stability across IoU thresholds. As shown in Section 3.1, when the IoU threshold increased from 0.5 to 0.75, YOLOv8-BS’s color detection AP remained robust at 73.2%, and spot detection AP was 86.7%, with F1-score declines of only 11.2 and 12.8 percentage points, respectively. In contrast, YOLOX and Faster R-CNN showed larger drops (e.g., YOLOX’s color AP fell from 58.2% to 43.8%), indicating weaker adaptability. This stability is crucial for real-world applications, where environmental variations (e.g., lighting, occlusion) may necessitate higher confidence thresholds [64,65].

Interestingly, all models performed better in spot detection than color detection, likely due to the distinct boundaries of spots compared to the subtle gradations of colors, which require finer feature extraction. YOLOv8-BS’s ability to achieve a high AP (86.8%) in color detection underscores its effectiveness in addressing this challenge, outperforming other YOLO-series models and Faster R-CNN. The confusion matrices in Figure 6 further validate its classification accuracy, with 87.6% for color detection and 92.3% for spot detection, despite minor misclassifications in edge cases. These edge cases mostly involve samples with ambiguous phenotypes, and their misclassification is mainly attributed to the following: in color detection, misjudgments commonly occur in samples in color transition states (e.g., bamboo shoots with hues between two typical colors) or samples with unstable color features caused by lighting conditions (especially low-saturation samples). This is partly due to the insufficient number of extreme cases in the training data, which limits the model’s ability to fully learn rare phenotypes. Future studies can improve the model’s robustness by expanding samples of transitional colors and extreme spot morphologies or introducing attention mechanisms to enhance the model’s capability to capture subtle features. Additionally, integrating technologies such as hyperspectral imaging to acquire multi-dimensional phenotypic data may provide new solutions for identifying ambiguous phenotypes.

Compared to recent plant phenotyping studies, YOLOv8-BS’s performance is highly competitive. For instance, De Nart, Gardiman, Alba, Tarricone, Storchi, Roccotelli, Ammoniaci, Tosi, Perria and Carraro [66] reported high-precision grape color classification using an improved ResNet model, while Yin, Li, Li and Yi [67] achieved 95% accuracy in citrus disease spot detection with MobileNetV3. YOLOv8-BS’s ability to handle both color and spot detection with APs exceeding 86% and 96% demonstrates its versatility and efficiency, positioning it as a significant advancement in bamboo shoot phenotyping.

### 4.2. Architectural Advantages of YOLOv8-BS

The superior performance of YOLOv8-BS in detecting bamboo shoot sheath colors and spots, as evidenced in Section 3, is driven by its optimized architecture, tailored for the complexities of phenotypic trait detection. Built on the YOLOv8s framework, the model balances accuracy and computational efficiency, making it ideal for field-based phenotyping applications [54]. The backbone of YOLOv8-BS incorporates the C2f (Cross-Stage Partial Fusion) module, an advancement of the CSPNet framework, which enhances feature extraction by integrating features across network stages, improving depth and expressiveness while minimizing computational costs [68]. The RepConv (Re-parameterizable Convolution) module further strengthens nonlinear modeling during training through a multi-branch structure, consolidating into a single branch at inference to optimize speed and efficiency [56]. These components enable YOLOv8-BS to capture intricate details, such as subtle color gradients and small spotting patterns, critical for accurate bamboo shoot phenotyping.

The neck module facilitates robust feature aggregation through three key components: the Spatial Pyramid Pooling Fast (SPPF) module, which employs multi-scale pooling (e.g., 5 × 5, 9 × 9 kernels) to capture features across varying sheath sizes; the Probabilistic Anchor Assignment (PAA) module, which optimizes sample selection to enhance training efficiency [58]; and the Path Aggregation Network (PAN), which fuses low-level spatial and high-level semantic features via bidirectional pathways, improving detection robustness [69]. This multi-scale fusion is particularly effective for handling the diverse morphologies of bamboo shoot sheaths in complex field conditions. The anchor-free detection head of YOLOv8-BS directly predicts target center coordinates, bounding box dimensions, and class probabilities, eliminating the need for predefined anchor boxes. This design simplifies training, enhances stability, and improves generalization across diverse phenotypes [42]. The Complete IoU (CIoU) loss function further refines bounding box accuracy by accounting for overlap, aspect ratio, and center distance, addressing the irregular shapes of bamboo shoot sheaths [70]. These architectural choices collectively contribute to the model’s high precision and recall, as evidenced by its performance metrics (Section 3.1). Compared to traditional two-stage detectors like Faster R-CNN, which are computationally intensive due to region proposal networks [71], YOLOv8-BS’s single-stage architecture offers superior speed and efficiency. While YOLOv7 and YOLOv5 share similar principles, YOLOv8-BS’s advanced modules (e.g., C2f, RepConv) provide enhanced feature extraction, leading to higher accuracy in detecting bamboo shoot traits. In contrast, traditional machine learning methods, such as Support Vector Machines and Random Forests, require manual feature engineering and struggle with complex backgrounds [72], underscoring YOLOv8-BS’s automated feature learning advantage. The architectural innovations of YOLOv8-BS position it as a state-of-the-art solution for bamboo shoot phenotyping, offering a robust framework for precision agriculture and botanical research applications.

### 4.3. Broader Implications and Future Directions

The YOLOv8-BS model’s high accuracy and efficiency in detecting bamboo shoot sheath colors and spots, as demonstrated in Section 3, have significant implications for the bamboo industry and scientific research, paving the way for advancements in precision agriculture and plant phenotyping. In precision agriculture, accurate phenotyping is essential for optimizing crop management and enhancing yield quality. The colors and spotting patterns of bamboo shoot sheaths serve as critical indicators of variety, health, and market value [21]. YOLOv8-BS enables automated, high-throughput phenotyping, streamlining processes such as variety classification, disease detection, and quality grading. For example, farmers can leverage the model’s high precision (e.g., 90.1% for spot detection) to assess bamboo shoot health rapidly, identifying stress or disease indicators early for timely interventions. This capability supports efficient harvesting and market grading, enhancing the economic viability of bamboo cultivation [7]. From a scientific perspective, YOLOv8-BS facilitates detailed phenotypic analysis, crucial for understanding the genetic basis of bamboo shoot traits. By quantifying sheath colors and spots, researchers can explore the genetic diversity of *C. utilis* bamboo shoots. It should be explicitly stated that the current research findings have only been validated for *C. utilis* from Jinfo Mountain, and this premise simultaneously defines the applicable boundaries of the model at the present stage. The model’s ability to process high-resolution images in complex field conditions makes it a valuable tool for genetic studies, where traditional manual methods are labor-intensive and error-prone [10]. Despite its strengths, YOLOv8-BS has limitations that warrant further exploration. Its training on *C. utilis* from Jinfo Mountain may limit generalizability to other bamboo species or environments. Future research could expand the dataset to include diverse species and growth conditions, enhancing the model’s adaptability. Additionally, integrating environmental data could provide insights into phenotypic variations, enabling more comprehensive predictive models [73]. Refining fine-grained classification, such as distinguishing subtle color variations, may require advanced feature extraction or hybrid models combining YOLOv8-BS with other architectures. Developing real-time detection systems could further revolutionize field monitoring, offering continuous phenotyping for large-scale plantations.

## 5. Conclusions

This study introduces the YOLOv8-BS model for the automated phenotyping of *C. utilis* bamboo shoots. The model targets critical traits such as sheath colors and spots, which are essential for variety classification and health assessment. The model achieved average precision values of 86.8% and 96.1% for color and spot detection, respectively, with F1-scores of 84.6% and 91.1%. These results outperform those of YOLOv7, YOLOv5, YOLOX, and Faster R-CNN. Its stability across IoU thresholds highlights its reliability for precision agriculture. The model’s success stems from its optimized architecture featuring the C2f module for enhanced feature extraction, SPPF and PAN for multi-scale fusion, and an anchor-free detection head for improved accuracy. This addresses the limitations of traditional methods. These advancements enable high-throughput phenotyping, supporting quality grading, genetic diversity studies, and breeding programs. Future efforts should expand the dataset for broader applicability and explore real-time detection. YOLOv8-BS sets a new standard for bamboo shoot phenotyping, advancing sustainable agriculture and research.

## Figures and Tables

**Table 1:** Detection performance of different models (color).

Model	Threshold = 0.5				Threshold = 0.75			
	P/%	R/%	F1/%	AP/%	P/%	R/%	F1/%	AP/%
YOLOv8-BS	85.9	83.4	84.6	86.8	79.3	68.3	73.4	73.2
YOLOv7	73.6	74.5	74.0	75.4	65.1	71.4	68.1	62.2
YOLOv5	70.6	69.8	70.2	71.2	68.5	65.2	67.1	52.6
YOLOX	61.0	66.9	63.6	58.2	51.2	57.6	53.9	43.8
Faster R-CNN	67.3	56.5	59.6	48.3	59.1	46.5	52.0	31.9

**Table 2:** Detection performance of different models (spots).

Model	Threshold = 0.5				Threshold = 0.75			
	P/%	R/%	F1/%	AP/%	P/%	R/%	F1/%	AP/%
YOLOv8-BS	90.1	92.5	91.1	96.1	73.5	83.8	78.3	86.7
YOLOv7	72.3	73.6	72.9	78.6	63.9	66.0	64.9	72.1
YOLOv5	67.0	64.8	65.9	74.6	58.6	57.2	57.9	68.1
YOLOX	60.3	71.9	63.6	53.4	60.0	65.3	61.4	49.8
Faster R-CNN	49.5	58.8	53.7	49.2	41.3	51.2	45.7	42.7

## Data Availability

The data mentioned in this paper are available on request from the corresponding author.
